# Laparoscopic Sigmoidectomy for Sigmoid Cancer With Persistent Descending Mesocolon: Anatomical Characteristics and Technical Tips

**DOI:** 10.7759/cureus.27942

**Published:** 2022-08-12

**Authors:** Sho Fujiwara, Kenji Kaino

**Affiliations:** 1 Department of Surgery, Iwate Prefectural Chubu Hospital, Kitakami, JPN

**Keywords:** rare anatomy, laparoscopic colorectal surgery, colorectal cancer, laparoscopic technique, persistent descending mesocolon

## Abstract

Although rare, persistent descending mesocolon (PDM) is an anatomical anomaly that carries potential risks for laparoscopic colorectal surgery. Impaired blood circulation of the reconstructed colon is especially risky during surgery. We report a case of sigmoid cancer with PDM, in which the patient underwent laparoscopic sigmoidectomy.

A 52-year-old man diagnosed with sigmoid cancer was referred to our hospital. PDM was identified with preoperative enhanced-contrast computed tomography, which revealed the sigmoid colon located in the right lower quadrant and a bear-claw inferior mesenteric artery (IMA). Preoperative examination showed cT1N0M0 stage I (Union for International Cancer Control {UICC} eighth). We were not able to identify the branches of IMA after the medial-to-lateral approach. We divided the mesentery and marginal artery and the main branches from IMA extracorporeally prior to lymphadenectomy. Each oral and anal side was dissected without touching the tumor. Then, we marked the line for lymphadenectomy using the dissected line of mesentery as an intracorporeal landmark. Pathological findings showed pT1N0M0 stage I (UICC eighth edition). The patient was discharged without complications.

Using this approach and the preoperative recognition of PDM, we performed laparoscopic sigmoidectomy with lymphadenectomy for early-stage PDM case successfully and safely. Our mesocolon dissection-first approach could be a feasible and safer approach for early-stage sigmoid cancer.

## Introduction

Persistent descending mesocolon (PDM) is a rare anatomical anomaly (2.1-2.3%) caused by the failure of the descending mesocolon to fuse with the posterior abdominal wall peritoneum during the embryonic period [1,2]. This anomaly can cause severe adhesion of the colon and mesentery with the surrounding structures and can lead to the following conditions: a midline-shift of the descending colon, the sigmoid colon located in the abdominal right lower quadrant (RLQ), shortened mesentery, and a bear-claw inferior mesenteric artery (IMA) [2-5]. Because it is rare and unfamiliar, case reports and clinical studies of laparoscopic surgery for colorectal cancer with PDM are limited, and standardization of surgical procedures is difficult to establish. Additionally, the characteristic anatomy makes safe laparoscopic surgery far-fetched. In fact, previous reports suggested that laparoscopic sigmoidectomy carries a high risk of longer operative time and more bleeding [4,6]. The present study aimed to evaluate the feasibility of our mesocolon dissection-first approach utilizing the mesocolon’s anatomical characteristics. Here, we report a case of laparoscopic sigmoidectomy for sigmoid cancer with PDM based on the above-mentioned features and demonstrate a technical tip as a salvage approach.

## Case presentation

**Figure 1:**
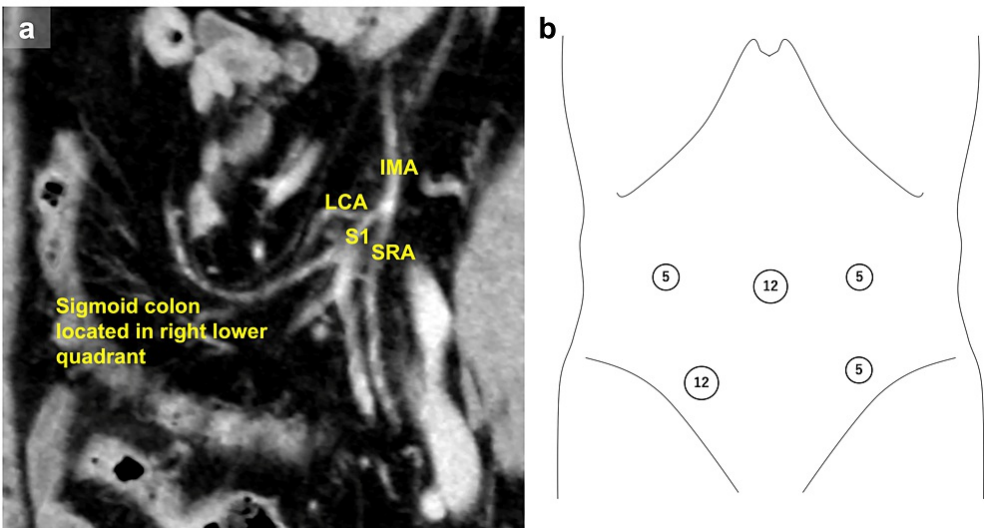
Preoperative enhanced-contrast computed tomography and port placement for laparoscopic sigmoidectomy. (a) Enhanced-contrast computed tomography revealed the sigmoid colon located in the right lower quadrant and a bear-claw inferior mesenteric artery; inferior mesenteric artery (IMA), left colic artery (LCA), first branch of the sigmoid artery (S1), and superior rectal artery (SRA). (b) Port placement for laparoscopic sigmoidectomy. The main operator switched the left side of the patient depending on the tumor location. We used the umbilical port for extracorporeal approach.

A 52-year-old man diagnosed with sigmoid cancer was referred to our hospital. PDM was identified preoperatively with enhanced-contrast computed tomography (CT), which revealed a midline-shift of the descending colon, the sigmoid colon located in the RLQ, shortened mesentery, and a bear-claw IMA (Figure 1, panel a). Preoperative examination showed cT1N0M0 stage I (UICC eighth edition).

**Figure 2:**
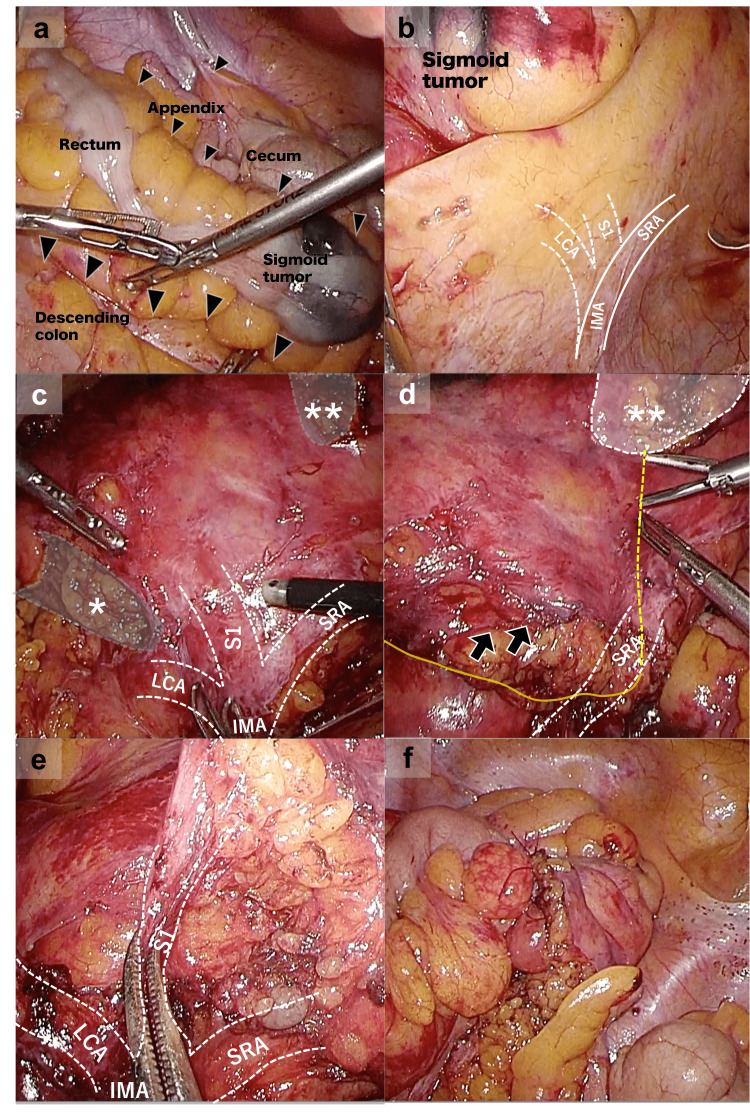
Intraoperative findings and procedures. The images are showing (a) dissecting the adhesions (arrowheads) of the sigmoid colon and mesentery in the right lower quadrant. (b) Identified the inferior mesenteric artery (IMA), first branch of the sigmoid artery (S1), and superior rectal artery (SRA). (c) The mesentery and marginal arteries were extracorporeally divided as far as possible prior to the dissection and division of S1. Each oral (*) and anal (**) side were dissected without touching the tumor. (d) Marking the line for lymphadenectomy using the dissected line of mesentery (**) as an intracorporeal landmark. (e) Lymphadenectomy and division of the S1. (f) Resection of the tumor and extracorporeal anastomosis.

The patient underwent a laparoscopic sigmoidectomy and lymphadenectomy. The port placement used in this procedure is shown in Figure 1, panel b. First, the main operator stood on the left side of the patient using left-sided positioning. We evaluated and dissected the adhesions of the sigmoid colon and mesentery and linearized the descending colon to the rectum and planarized the left-side mesentery (Figure 2, panel a). Second, the main operator then switched the side of the patient from left to right using right-sided positioning. We identified the IMA and divided proximal to lateral peritoneal attachments, also known as a medial-to-lateral approach, carrying the ureter and nerves because the PDM cases have no dissectible layer. In PDM cases, peritoneal attachments are inflammatory adhesions instead of the embryonic lateral peritoneal fusion (mobilization step; Figure 2, panel b). In this step, because mesentery was thick with adhesion and chronic inflammation, we were not able to identify the left colic artery (LCA) and first branch of sigmoid artery (S1). Third, we planned an extracorporeal anastomosis depending on the tumor location. Considering the bear-claw IMA and the dissection of the first branch of the sigmoid artery, which is necessitated by lymphadenectomy, we had to preserve the IMA and sigmoid artery. Thus, the mesentery and marginal arteries were extracorporeally divided as far as possible prior to the dissection and division of the superior rectal artery (SRA) (mesocolon step; Figure 2, panel c). Each oral and anal side was dissected without touching the tumor. Fourth, we performed lymphadenectomy and division of the SRA branches using the dissected line of mesentery as an intracorporeal landmark (lymphadenectomy step, Figure 2, panels d and e). Finally, we resected the tumor and performed an extracorporeal anastomosis (Figure 2, panel f). The operative time was 230 min, and the blood loss was 38 mL. The patient was discharged on postoperative day nine without complications. Pathological findings showed pT1N0M0 stage I (UICC eighth edition). The patient is alive without recurrence 28 months after surgery.

## Discussion

Laparoscopic sigmoidectomy for sigmoid cancer is an advanced but well-established minimally invasive surgical technique [7]. However, sigmoid cancer with PDM is rare and associated with some risks, thus, standardization of surgical procedures is lacking [4,6].

A retrospective study that evaluated the risks of laparoscopic surgery for colorectal cancer with PDM suggested that a high risk exists when operative time and blood loss are increased in laparoscopic sigmoidectomy and high anterior resection, but not in low anterior resection [4]. This is due to careful colorectal blood flow preservation in sigmoidectomy and high anterior resection compared with low anterior resection, considering the bear-claw IMA, marginal artery, and shortened mesentery; extracorporeal anastomosis should preserve much more arteries for blood flow of both oral and anal sides [6].

When the conventional procedure is difficult because of the PDM, our mesocolon dissection-first approach can be applicable for laparoscopic sigmoidectomy cases with PDM limited to stage I. We divided the mesentery, marginal artery, and the main branches from IMA extracorporeally prior to dissection of feeding artery and lymphadenectomy without blood flow impairment of the reconstructed colon. In the cases of colorectal cancer with PDM, a retrospective study suggested that 70% of the inferior mesenteric vein (IMV) and left colic artery were divided extracorporeally, but 5.7% of them were divided extracorporeally [5]. Therefore, we can dissect the mesentery extracorporeally without touching the tumor. However, extracorporeal mesentery dissection prior to artery division is often difficult in non-PDM cases because of the sigmoid colon’s mobility [8]. The shortened mesentery in PDM in a conventional approach carries a higher risk of damage to the essential arteries that maintain the remnant blood flow [6,9,10]. Retrospective radiological findings suggested that the median distance between the IMA and the IMV was 14.8 mm and the IMV and the descending colon was 17.2 mm in PDM cases, compared with 23.0 mm and 90.0 mm in non-PDM cases, respectively [5]. Furthermore, in a typical laparoscopic surgery for colorectal cancer, considering the safeness and oncological aspects, lymphadenectomy and division of vessels were often performed prior to mesenteric dissection with the no-touch isolation technique using conventional medial-to-lateral approach [7]. But the impact of conventional medial approach on overall survival is still controversial [11-13]. These data support the extracorporeal mesocolon dissection-first approach as a safer and more applicable approach than the conventional one.

Furthermore, preoperative evaluation for the identification of PDM is also an essential step. Radiological features such as right-shift of the IMA, midline-shift of the descending colon, sigmoid colon located in RLQ, shortened mesentery (between the IMA and IMV or between the IMV and descending colon), and bear-claw IMA have been reported [2,5]. In this case, we identified PDM with atypical artery branches and the location of the sigmoid colon in preoperative CT. A routine preoperative evaluation is sufficient for diagnosis; however, understanding the risk and features of PDM cases is crucial, enabling us to plan the approach in accordance with the PDM while minimizing the complications and maximizing safety [5,9,14,15].

Our study has a few limitations. It does not evaluate the postoperative outcomes and long-term outcomes compared with the conventional approach. In fact, laparoscopic surgery with PDM cases leads to various postoperative complications. For example, anastomotic stenosis and leakage are associated with blood flow impairment of the reconstructed colon [4,6,10]. Our approach may help preserve adequate blood flow during surgery due to extracorporeal identification of the important branches and minimization of the dissection area of lymphadenectomy for early-stage cases. Even if this approach is easier and safer, we did not evaluate the short-term outcomes and feasibility of the approach compared with that of the conventional approach. Two Japanese high-volume centers have reported retrospective studies about PDM [4,5]. The mean operative time and blood loss were 217.7 min and 32.3 mL, respectively [4]. Although a statistical comparison cannot be analyzed, our outcome is feasible compared with their data. However, a higher number of cases is required to evaluate the long-term oncological outcomes.

## Conclusions

Although rare, laparoscopic sigmoidectomy for sigmoid cancer with PDM has not been well established and detailed surgical procedures have not been described. Mesocolon dissection-first approach could be a feasible and safer approach for early-stage sigmoid cancer with PDM by utilizing the anatomical characteristics to our advantage as a salvage approach.
